# Unmet supportive care needs among informal caregivers of patients with head and neck cancer in the first 2 years after diagnosis and treatment: a prospective cohort study

**DOI:** 10.1007/s00520-023-07670-1

**Published:** 2023-04-13

**Authors:** Kira S. van Hof, Arta Hoesseini, Maarten C. Dorr, Irma M. Verdonck - de Leeuw, Femke Jansen, C. Réne Leemans, Robert P. Takes, Chris H. J. Terhaard, Robert J. Baatenburg de Jong, Aniel Sewnaik, Marinella P. J. Offerman

**Affiliations:** 1 Department of Otorhinolaryngology and Head and Neck Surgery, Erasmus MC Cancer Institute, Dr. Molewaterplein 40, 3015 GD Rotterdam, The Netherlands; 2 Department of Otolaryngology and Head and Neck Surgery, Amsterdam UMC, Location Vrije Universiteit Amsterdam, Cancer Center Amsterdam, Cancer Center Amsterdam, De Boelelaan 1117, Amsterdam, The Netherlands; 3 Cancer Center Amsterdam, Treatment and Quality of Life, Amsterdam, The Netherlands; 4 Department Clinical, Neuro and Developmental Psychology, Vrije Universiteit Amsterdam, Van Der Boechorststraat 7-9, Amsterdam, The Netherlands; 5 Amsterdam Public Health, Mental Health, Amsterdam, The Netherlands; 6 Department of Otolaryngology and Head and Neck Surgery, Radboud University Medical Center, Nijmegen, The Netherlands; 7 Department of Radiation Oncology, University Medical Center, Utrecht, The Netherlands

**Keywords:** Supportive care needs, Informal caregivers, Caregiver burden, Psychological distress, Quality of life, Head and neck neoplasms

## Abstract

**Objective:**

Informal caregivers of head and neck cancer (HNC) patients have a high caregiver burden and often face complex practical caregiving tasks. This may result in unmet supportive care needs, which can impact their quality of life (QoL) and cause psychological distress. In this study, we identify caregivers’ unmet needs during long-term follow-up and identify caregivers prone to unmet supportive care needs.

**Methods:**

Data were used from the multicenter prospective cohort study NETherlands QUality of life and Biomedical cohort studies In Cancer (NET-QUBIC). The unmet supportive care needs, psychological distress, caregiver burden, and QoL were measured for 234 informal caregivers and their related patients at baseline, 3, 6, 12, and 24 months after. Mixed effect models for repeated measurements were used.

**Results:**

At baseline, most caregivers (70.3%) reported at least one unmet supportive care need, with most of the identified needs in the “healthcare & illness” domain. During the follow-up period, caregivers’ unmet needs decreased significantly in all domains. Nevertheless, 2 years after treatment, 28.3% were still reporting at least one unmet need. Financial problems were increasingly associated with unmet needs over time. Furthermore, caring for a patient who themselves had many unmet needs, an advanced tumor stage, or severe comorbidity was associated with significantly more unmet needs in caregivers.

**Conclusions:**

The current study shows the strong likelihood of caregivers of HNC patients facing unmet supportive care needs and the interaction between the needs of patients and caregivers. It is important to optimally support informal caregivers by involving them from the start when counseling patients, by providing them with relevant and understandable information, and by referring vulnerable caregivers for (psychosocial) support.

**Supplementary Information:**

The online version contains supplementary material available at 10.1007/s00520-023-07670-1.

## Introduction

Head and neck cancer (HNC) is an aggressive type of cancer that causes approximately 300,000 deaths worldwide each year [1, 2]. In many cases, HNC and the associated treatment causes an impaired body image and major comorbidities, such as difficulties with eating, swallowing, and speaking [3]. As such, HNC can have a notable impact on patients’ quality of life (QoL) and cause psychological distress [4–6]. The impact of HNC and its treatment also extends to a patient’s direct environment including spouses, children, and other informal caregivers [7–10]. Informal caregivers in this paper are viewed as a patient’s primary emotional support person, and they often face complex practical caregiving tasks, like help the patient taking care of a tracheostomy [9]. Previous research shows that informal caregivers can experience even higher levels of anxiety than the patients they care for [8, 11, 12]. Both patients and their caregivers have to deal with the consequences of the disease, which may result in unmet supportive care needs for both [13]. The most often used description of “unmet needs” is the discrepancy between received support and the support needed to achieve optimal wellbeing [14].

There is cumulative evidence that supportive care helps in coping with the effects of disease and treatment and reduces psychological distress [15, 16]. While most supportive care programs focus on patients [16], informal caregivers experience their own supportive care needs in different domains: “emotional & relational,” “healthcare & illness,” “practical,” and “work & social” [16]. Previous cross-sectional studies indicate that the unmet supportive care needs of caregivers of HNC patients can lead to an increased caregiver burden, emotional distress, and a reduced QoL [13, 17]. The psychological distress felt by informal caregivers is related to the HNC patient’s QoL and psychological distress [8, 18]. Caregivers’ unmet needs can undermine the support they can provide to the patient and may even lead to psychological or medical problems in the caregivers themselves [17]. The fact that unmet supportive care needs are related to psychological distress, and that informal caregivers’ psychological distress is related to patients’ functioning, underlines the importance of offering supportive care to caregivers [8, 18]. Despite this, there is a lack of longitudinal long-term follow-up studies regarding the unmet needs of HNC caregivers [19, 20]. In the only longitudinal study identified, Hung et al. described the unmet needs of 142 HNC caregivers during the first 3 months following patient discharge [21]. They found that unmet supportive care needs peaked one week after discharge, but had significantly decreased three months later [21]. The longer-term trends in unmet supportive care needs during the cancer survivorship phase remain unclear. Our hypothesis is that supportive care needs will change throughout the follow-up trajectory: After diagnosis, the main focus will be on surviving, while, subsequently, the patient and their direct environment need to adapt to the new situation.

The aims of this study were to (1) assess the unmet supportive care needs of informal caregivers of HNC patients from diagnosis through two years of follow-up; (2) evaluate the relationship between the unmet needs of HNC patients and their informal caregivers; and (3) identify variables associated with the unmet supportive care needs of caregivers of HNC patients.

## Material and methods

### Setting and participants

This study used data collected between March 2014 and June 2018 in the ongoing “Netherlands Quality of life and Biomedical Cohort in HNC” (NET-QUBIC) multicenter prospective cohort study [22]. Participants from five participating medical centers in the Netherlands were included. Patients were recruited by a local researcher after diagnosis. All the participating HNC patients were asked if their primary informal caregiver would be willing to cooperate in our study. ‘Informal caregiver’ was defined as a relative or friend providing assistance to a patient.

### Exclusions and eligibility

Patients were eligible if they were diagnosed with a new squamous cell carcinoma in the head and neck region (oral cavity, oropharynx, hypopharynx, or larynx, or neck lymph node metastasis of an unknown primary tumor), with an intention to undergo curative treatment. Furthermore, patients had to be older than 18 years, and caregivers and patients had to be able to read and write Dutch. Exclusion criteria were as follows: patients with previously treated tumors or caregivers or patients with severe psychiatric disorders such as Korsakoff syndrome, schizophrenia, or severe dementia. Ethical approval was obtained through the coordinating center (VU University Medical Center Amsterdam: (2013.301(A2018.307)-NL45051.029.13) and in the corresponding center (Erasmus Medical Center Rotterdam). All participants provided written informed consent. Further details about the inclusion process for the NET-QUBIC study can be consulted elsewhere [22].

## Measures

Both informal caregivers and patients completed questionnaires distributed and returned through the postal mail. Data were collected at five points: baseline (after diagnosis and before start of treatment) and then 3, 6, 12, and 24 months after treatment. An electronic Case Report Form (eCRF) was used (OpenClinica) and additional patient clinical information was derived from their medical records.

Demographic characteristics were collected using the eCRF and self-reported questionnaires. Performance status was established using the World Health Organization (WHO) performance status, ranging from fully active (0) to dead (5) [23]. Furthermore, comorbidity was evaluated based on the Adult Comorbidity Evaluation-27 (ACE-27) scale, which provides a severity score ranging from 0 to 3 [24].

The supportive care needs of informal caregivers were identified using the 45-item Supportive Care Needs Survey-Partners and Caregivers (SCNS-P&C45) [16]. Each item is scored on a five-point scale, indicating: no needs (1: not applicable, 2: needs fulfilled), low (3), moderate (4), or high unmet needs (5). Following Girgis et al., both moderate and high unmet needs were considered ‘unmet’ for the purposes of our study [16, 26]. Four domains are identified: (1) “emotional & relational” (i.e., “Looking after your own health”), (2) “healthcare & illness” (i.e., “obtaining the best medical care for the patient”), (3) “practical” (i.e., “finding out about financial support and government benefits”), and (4) “work & social” (i.e., “handling the topic of cancer in social situations or at work”). Scores for each domain range from 0 to 100, with higher scores indicating greater unmet needs.

Supportive care needs of patients were identified using the 34-item Supportive Care Needs Survey-Patient (SCNS-SF34) and the Supportive Care Needs Survey-Head and Neck Cancer (SCNS-HNC) [26, 27]. Similar to the caregiver questionnaire, the SCNS-SF34 and the SCNS-HNC together contain 45 supportive care needs. A total score for unmet needs was calculated as the number of questions answered with either moderate or high unmet needs.

Psychological distress was measured using the Hospital Anxiety and Depression Scale (HADS) [28]. This questionnaire has two subdomains (anxiety and depression) both containing seven items. The total scores for each domain can vary from 0 to 21, with higher scores reflecting greater anxiety or more depression symptoms [29].

Global quality of life (QoL), physical functioning, and social functioning were measured with the European Organization for Research and Treatment of Cancer Quality of Life Questionnaire (EORTC-QLQ-C30) [30]. Each subscale results in a score between 0 and 100, with a higher score indicating healthier functioning. These subscales were chosen as the most relevant after consultation of a group of different healthcare experts, as a head and neck surgeon, psychologists, and senior researchers.

Caregiver burden was determined using the caregiver reaction assessment (CRA) [31]. This questionnaire comprises five caregiver reactions: (1) a positive impact on self-esteem, (2) a negative impact on disrupted schedule, (3) family support, (4) financial problems, and (5) health problems. Each domain provides a score ranging from 0 to 5, where a higher score on self-esteem (1) indicates a positive caregiver reaction and a higher score on the remaining domains (2–5) reflect negative effects of caregiving.

### Statistical analysis

Data analyses were performed in R [32]. Descriptive statistics were used to describe the study sample and the single “unmet needs” items. The effect of time on caregivers’ unmet supportive care needs was assessed using linear mixed model analyses. To estimate which variables were associated with caregivers’ unmet supportive care needs in each of the four SCNS-P&C45 domains, four models were tested and fitted that included caregiver-related variables (gender, age, education level, caregiver type, baseline scores on the HADS and EORTC subdomains) and patient-related variables (tumor stage, WHO-status, comorbidity, number of unmet supportive care needs at baseline) as fixed effects and time as a random effect. Mixed model analyses were used as they permit for missing values in the repeated data. In addition, the JointAI package was used to impute missing values in the covariates so that all available data could be used [33]. A two-sided *P* value of less than 0.05 was set as the criterion for statistical significance.

## Results

### Demographic characteristics

A total of 262 pairs of informal caregivers and patients met the inclusion criteria. Nine caregivers dropped-out for varying reasons before the baseline assessment (Appendix 1). Another 19 informal caregivers did not complete any of the PROMs and were therefore excluded from the analysis. Consequently, a total of 234 informal caregiver-patient pairings were included. The baseline characteristics of this sample are presented in Table 1.

**Table 1:** Descriptive characteristics of informal caregivers and patients

	Patients (*N* = 234) Mean (SD) Frequency (%)	Total number missing (%)	Caregivers (*N* = 234) Mean (SD) Frequency (%)	Total number missing (%)
Age, years	63.6 (9.6)	0 (0%)	59.4 (11.3)	0 (0%)
Age, range	35–85	0 (0%)	19–88	0 (0%)
Gender		0 (0%)		0 (0%)
Male	177 (75.6%)		64 (27.4%)	
Female	57 (24.4%)		170 (72.7%)	
Caregiver type Spouse Daughter/son Other			199 (85.0%) 26 (11.1%) 9 (3.8%)	0 (0.0%)
Education level		15 (6.4%)		13 (5.5%)
Low Intermediate High	83 (35.5%) 62 (26.5%) 74 (31.6%)		82 (36.9%) 62 (27.9%) 78 (35.1%)	
Tumor site		0 (0%)		
Oral cavity Oropharynx Hypopharynx Larynx Unknown primary	68 (29.1%) 77 (32.9%) 13 (5.6%) 67 (28.6%) 9 (3.8%)			
Disease stage I II III IV	54 (23.1%) 43 (18.4%) 37 (15.8%) 100 (42.7%)	0 (0%)		
WHO performance		0 (0%)		
0	176 (75.2%)			
I–II	58 (24.8%)			
Comorbidity		16 (6.8%)		
None	65 (29.8%)			
Mild	85 (39.0%)			
Moderate	44 (20.2%)			
Severe	24 (11.0%)			

## Informal caregivers unmet supportive care needs

### Total unmet needs

**Fig. 1:**
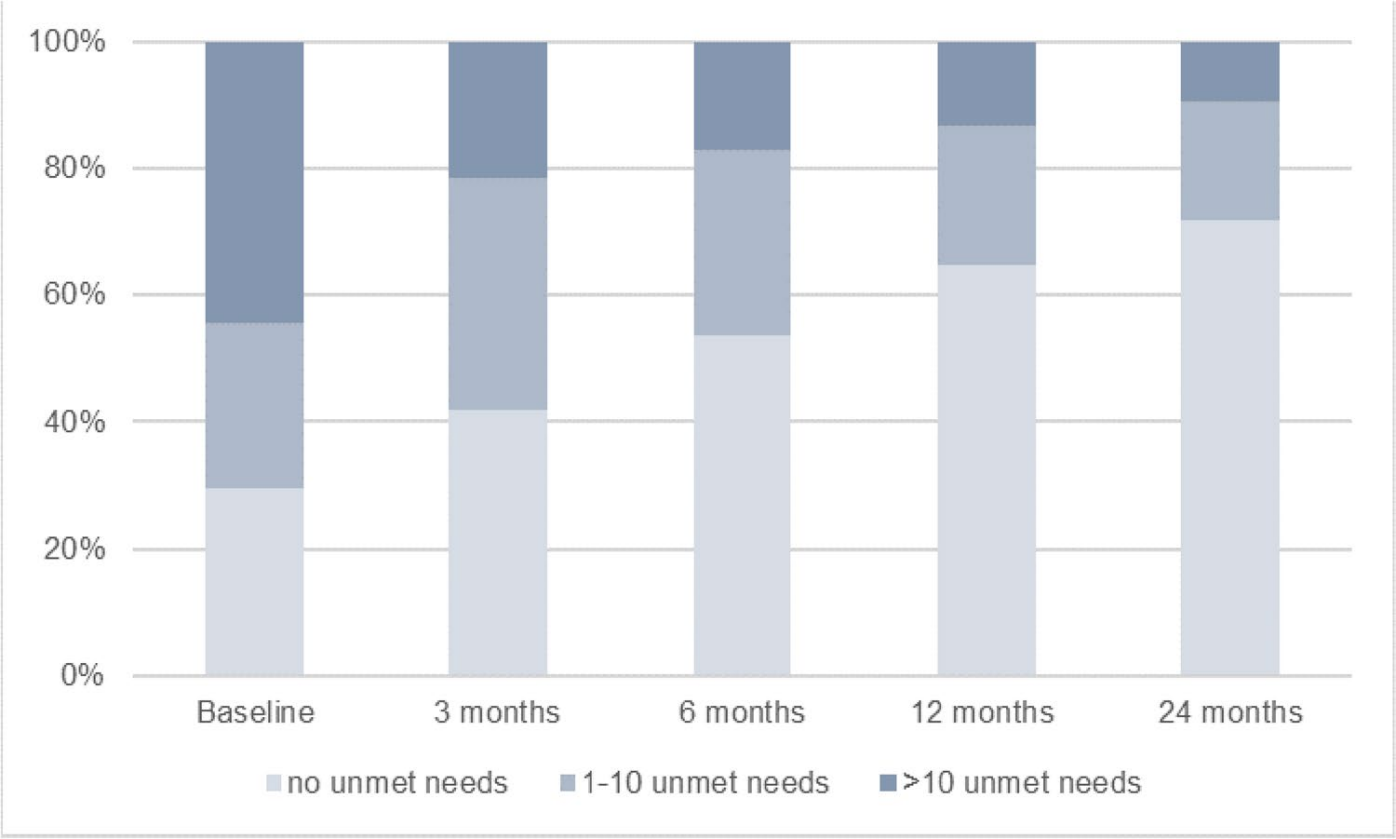
The percentage of informal caregivers with no, moderate or high unmet supportive care needs over time. Supportive care needs were considered “unmet” when caregivers reported a moderate to high need (4 and 5) on that item on the Supportive Care Needs Survey-Partners and Caregivers (SCNS-P&C45) scale

In the baseline assessment, 56 caregivers (29.6%) reported no unmet needs, 49 (25.9%) reported between 1 and 10 unmet needs, and 84 (44.4%) reported having more than 10 unmet needs. During the follow-up assessments, the number of caregivers with unmet needs decreased over time (Fig. 1). Two years after treatment, most caregivers (71.7%) reported no unmet needs, 24 (18.9%) 1–10 unmet needs, and 12 (9.4%) more than 10 unmet needs. On average, caregivers reported 10 (SD: 10.5) unmet needs at the baseline, 6 (9.7) three months, 5 (8.1) six months, 3 (6.9) twelve months, and 3 (7.0) twenty-four months after treatment.

### Unmet supportive care needs per domain

**Fig. 2:**
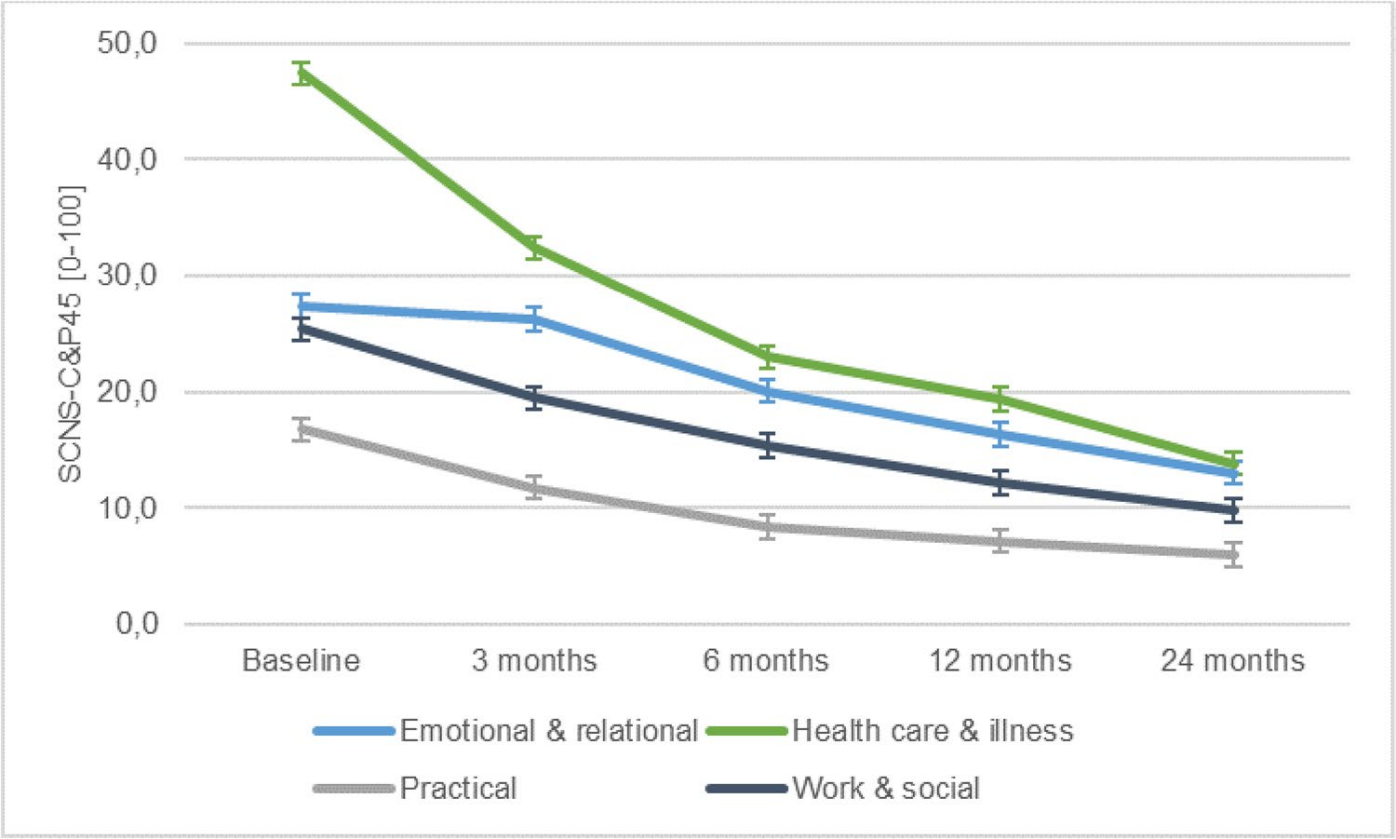
Caregivers mean supportive care needs over time per SCNS-P&C45 domain. The Supportive Care Needs Survey-Partners and Caregivers (SCNS-P&C45) consists of four domains: “emotional & relational” (i.e.. “looking after your own health”), “healthcare & illness” (i.e., “obtaining the best medical care for the patient”), “practical” (i.e., “finding out about financial support and government benefits”), and “work & social” (i.e., “handling the topic of cancer in social situations or at work”). Scores per domain range from 0 to 100, with higher scores suggesting higher unmet needs

At baseline, the highest scores in terms of unmet needs were in the “healthcare & illness related needs” domain (mean 47.4, SD 29.4). Next, in terms of unmet needs was the “emotional & relational needs” domain (mean 27.4, SD 21.0), followed by “work & social needs” (mean 25.4, SD 22.0), with the “practical needs” domain having the fewest unmet needs (mean 16.8, SD 22.4). A significant reduction in needs unmet was seen in all four domains during the follow-up assessments (*p* < 0.001) (Table 3). As can be seen in Fig. 2, the biggest reduction was seen in the “healthcare & illness” domain where, 6 months after treatment, the unmet needs had more than halved (mean 23.0). The difference between “healthcare & illness” needs and “emotional & relational” needs was significant during the first three measurement moments (*p* < 0.01), but no longer significant 12 and 24 months after treatment. The unmet needs in both the “practical needs” and the “work & social needs” domains remained significantly below the other two domains throughout the assessment period (*p* < 0.002).

### Single item unmet supportive care needs

In terms of single item unmet needs, the most reported items were all in the “healthcare & illness related” domain (Table 2). Both at the baseline and one year after treatment, “obtaining the best medical care for the person with cancer” was the most reported single item. In the intervening period, “reducing stress in the patient’s life” was the most frequently reported item three months after treatment and “making sure complaints regarding the patient’s care are properly addressed” the most common six months after treatment. In our final assessment, two years after treatment, the most frequent concern was that caregivers were not “Feeling confident that all the doctors are talking to each other to coordinate the patient’s care.”

**Table 2:** The most reported unmet individual item supportive care needs

SCNS-P&C items “in the last month, what was your level of need for help with…”	Domain	T0	M3	M6	M12	M24
Obtaining the best medical care for the patient	Healthcare and illness	**52%**	30%	20%	**18%**	8%
Being involved in the patient’s care, together with the medical team	Healthcare and illness	48%	29%	17%	14%	11%
Accessing information about the patient’s prognosis, or likely outcome	Healthcare and illness	48%	31%	15%	17%	8%
Ensuring there is an ongoing case manager to coordinate services	Healthcare and illness	48%	25%	17%	13%	11%
Making sure complaints regarding the patient's care are addressed	Healthcare and illness	48%	30%	**21%**	15%	11%
Feeling confident that all the doctors are talking to each other	Healthcare and illness	48%	27%	**21%**	14%	**13%**
Having opportunities to discuss your concerns with the doctors	Healthcare and illness	46%	28%	20%	15%	12%
Reducing stress in the patient’s life	Healthcare and illness	43%	**32%**	20%	16%	11%
Accessing local health care services when needed	Healthcare and illness	32%	19%	12%	12%	11%

Needs were defined as “unmet” when caregivers reported a moderate or high need (rated at 4 or 5) for an item on the Supportive Care Needs Survey-Partners and Caregivers (SCNS-P&C45). The table includes all items that were one of the top 5 reported needs at any one of the measurement points. The most reported item at each point is bolded

Two years after treatment, the “emotional & relational” domain was causing nearly as much concern as “healthcare & illness” needs (Fig. 2). The most reported single items under “emotional & relational” needs at this 2-year point were “looking after your own health, including eating and sleeping properly” (reported by 9.7% of caregivers), “adjusting to changes in the patient’s body” (9.1%), and “understanding the experience of the person with cancer” (8.3%). Nearly all single item needs, across all four domains, were most strongly felt in the first assessment (at baseline) and then declined with three exceptions where the items were most often reported three months after treatment: “managing concerns about the cancer coming back” (25.9%), “the impact that cancer has had on your relationship with the patient” (16.1%), and “addressing problems with your sex life” (9.9%). In these three cases, the highest need for help was reported three months after treatment.

## Variables associated with unmet supportive care needs in caregivers

The reported unmet supportive care needs were significantly associated with caregiver-related variables (i.e., baseline global QoL and financial problems) and with patient-related variables (i.e., tumor stage, comorbidity, patient’s unmet needs at baseline) (Table 3). A higher level of global QoL and having more financial problems were both associated with more unmet needs in the practical domain (*p* = 0.049 and *p* = 0.003). Compared to caregivers caring for a patient with tumor stage I, caregivers for patients with stage III or IV tumors reported more unmet needs across all four domains. Caring for a patient with severe comorbidity was particularly associated with having more needs in the “emotional & relational” domain. There was a positive association between patients who reported higher level of unmet supportive care needs at baseline and higher levels of caregiver needs in the “emotional & relational” (*p* = 0.008) and the “work & social” domains (*p* = 0.019) at all measurement moments.

**Table 3:** Variables associated with unmet supportive care needs in caregivers

Supportive care needs	Variable baseline		Estimate (95% CI)	*p* Value
Emotional and relational	Tumor stage (P) Comorbidity (P) Unmet needs (P)	I II III IV None Mild Moderate Severe	“ 1.56 (– 4.64–7.82) 9.01 (2.23–15.92) 5.27 (– 0.02–10.65) “ 5.36 (– 0.07–10.83) 7.52 (0.84–14.13) 3.93 (– 4.02–11.92) 0.45 (0.12–0.78)	0.627 0.010* 0.051 0.054 0.030* 0.333 0.008*
	Time (months)		– 0.58 (– 0.69 to – 0.48)	< 0.001*
Healthcare and illness	Tumor stage (P)	I II III IV	“ 6.43 (– 1.80–14.55) 10.09 (1.26–19.96) 9.83 (2.96–16.74)	0.126 0.024* 0.005*
	Time (months)		– 1.20 (– 1.38 to – 1.05)	< 0.001*
Practical	Global QoL (C) Financial problems (C)		0.17 (0.00–0.34) 4.04 (1.30–6.83)	0.049* 0.003*
	Tumor stage (P)	I II III IV	“ 4.50 (– 1.33–10.40) 8.68 (2.33–15.07) 6.04 (1.09–11.04)	0.131 0.006* 0.017*
	Time (months)		– 0.37 (– 0.50 to – 0.26)	< 0.001*
Work and social	Tumor stage (P) Unmet needs (P)	I II III IV	“ 3.49 (– 2.45–9.38) 6.48 (0.13–12.9) 4.33 (– 0.64–9.31) 0.40 (0.07–0.72)	0.245 0.045* 0.087 0.019*
	Time (months)		– 0.59 (– 0.71 to – 0.46)	< 0.001*

Linear mixed models were adjusted for gender, age, education level, caregiver type, tumor stage, WHO status, total unmet needs of patient (baseline), caregiver burden (baseline), anxiety caregiver (baseline), depression caregiver (baseline), physical functioning (baseline), and social functioning (baseline). C = caregiver, P = patient

## Discussion

Through this prospective multicenter cohort study, we have gained insight into the unmet supportive care needs of informal caregivers of HNC patients from baseline until two years after treatment. Most (70%) of the caregivers had unmet needs at baseline but these decreased significantly during the follow-up period. Nevertheless, two years after treatment, still more than one in four (28.3%) of the informal caregivers reported at least one unmet need. Both patient-related and caregiver-related variables were associated with these unmet supportive care needs. HNC patients’ and informal caregivers’ needs were interrelated, with patients’ total unmet needs associated with specific domains of unmet needs of informal caregivers during the follow-up period, a finding which is consistent with earlier studies [7, 34, 35]. Further, the results of this study add to the literature since there have previously been very few longitudinal studies that assess the changing supportive care needs of caregivers through diagnosis, treatment, and the subsequent trajectory [20].

The finding that unmet supportive care needs decrease over time is also consistent with earlier research. Girgis et al. also found that unmet needs reduced over time in a cohort of caregivers of cancer patients in general. They similarly found that one-third of the caregivers experienced at least one unmet need two years after treatment [36]. They reported a higher average unmet needs six months after treatment (mean 6.7, SD: 10.7) than in our cohort (mean 4.6, SD: 8.1); however, the numbers of unmet needs in their study one and two years after treatment were similar to our results. The difference between this result and our study results might has to do with the small samples size as only 60 HNC caregivers were included. Moreover, the informal caregivers in our study reported a higher average number of unmet needs 6, 12, and 24 months after treatment than caregivers of patients with other cancer types in the study of Girgis et al. (melanoma, colorectal, breast, and prostate carcinomas) [36].

In our study, the most unmet needs fell in the “healthcare & illness needs” domain, especially during the first 6 months following treatment. Previous cross-sectional studies addressing HNC caregivers similarly reported high percentages of caregivers with unmet needs regarding information in the “healthcare & illness” area [17, 37]. However, Hanly et al. came to a different conclusion, finding the most unmet needs in the “psychological & emotional” domain [13]. Due to the cross-sectional design of their study, it is hard to compare their results with ours as unmet supportive care needs decreased significantly over time and the differing domains saw various rates of decay in our cohort. Furthermore, findings from other qualitative studies support the high level of unmet “healthcare & illness” related needs and also point to the need to ensure that information is communicated in an understandable way [38–40]. These results also reflect those of Lambert et al., who concluded that many cancer caregivers did not get the information they needed on what to expect from the disease and treatment [15].

In our baseline assessment, more than half of the informal caregivers reported an unmet need in terms of “obtaining the best medical care.” However, given that the baseline measurement was taken before the start of treatment, it is understandable that caregivers had unmet needs in terms of obtaining the best medical care. This issue was not even one of the top 10 unmet needs reported in the cross-sectional study by Chen et al. where unmet needs were only measured after treatment had started [17]. The number of caregivers that reported unmet needs in terms of “feeling confident that all the doctors are talking to each other” was more stable over time. In our cohort, “managing concerns about the cancer coming back” had become the most common concern three months after treatment (25.9%). This is a similar finding to that of Balfe et al. (21% HNC caregivers reporting moderate or high unmet needs) [17]. Addressing other cancer types, Sklerova (2015) found a higher percentage of caregivers (44.6%) with moderate or high needs related to “managing concerns about the cancer coming back” [41]. A possible explanation for these differences is that their studies are older and that more information about the prognosis is nowadays made available. Furthermore, patients are today more involved in the decision-making process, which may give them a better feeling of being in control of the situation. Other items that were reported more frequently three months after treatment than initially were as follows: “the impact that cancer has had on your relationship with the patient” (16.1%) and “addressing problems with your sex life” (9.9%). This observation supports our hypothesis that, immediately after diagnosis, the main focus of caregivers is on getting a grip on the situation, while, as time moves on, both caregivers and patients together need to adapt to the new situation including their relationship and intimacy [7].

The unmet needs of caregivers were statistically associated with caregiver-related and patient-related variables. Caring for a patient with a high number of unmet supportive care needs, severe comorbidity, or with an advanced tumor stage was associated with caregivers reporting more unmet supportive care needs. Hodgkinson et al. similarly found that a more advanced disease stage was associated with higher needs in HNC caregivers [42]. In our cohort, no significant relationships were found between unmet needs and caregivers’ gender or age, a finding consistent with the review by Lambert et al. [15].

Consistent with other literature, we found a significant association between caregivers’ financial problems due to caregiving and unmet practical supportive care needs [43]. This association was not unexpected as the single items in the “unmet needs of the practical domain” are focused on finances, i.e., “finding out about financial support and government benefits.” However, contrary to our expectations, we also found a positive relationship between better global QoL and more unmet needs in the practical domain (*p* = 0.049).

### Strengths and limitations

A major strength of this study is the large multicenter cohort used with a prospective longitudinal design, giving insight into the unmet supportive care needs over time, from diagnosis through to two years after treatment. Moreover, the impact of caregiver-related variables and patient-related variables was estimated. By using linear mixed model analyses for repeated measures, we were able to use all the available information and did not have to exclude participants with missing data. Nevertheless, the response rate did decrease (to 68%) over the follow-up period, which could have biased our results (Appendix 1). Also, as the items of the SCNS-P&C45 and the SCNS-SF34 and SCNS-HNC scales do not fully correspond, it was not possible to compare the specific needs of caregivers and of HNC patients in depth. Finally, it is possible that our cohort is not fully representative of the overall HNC population as 75% of the included patients had a good performance stage (WHO 0) [23].

### Clinical implications and future perspectives

The unmet supportive care needs of informal caregivers of HNC patients change over time. Immediately after diagnosis, caregivers often experience feelings of uncertainty and loss of control [7], which may explain why the most unmet needs are linked with obtaining the best (organized) care and information about the prognosis or likely outcome. After treatment, when caregivers and patients have to adapt to their new lives beyond cancer, negative feelings may emerge that lead to other unmet supportive care needs (“emotional & relational needs”). Healthcare professionals should involve caregivers in the counseling and support they offer to patients. Structural screening and monitoring using standardized questionnaires at several intervals can be used to identify vulnerable caregivers that may benefit from additional psychosocial support [8]. Furthermore, optimizing the provided information may help to reduce caregivers’ feelings of uncertainty and loss of control (unmet “healthcare & illness needs”). In a recent review by Wang et al. [20], multiple experimental interventions were proposed to reduce unmet needs in HNC caregivers, varying from face-to-face workshops to information tools through DVDs and websites [20]. All the studies they included stated that having an opportunity to have contact with patients or informal caregivers that were experiencing a similarly trajectory was beneficial and reassuring [20]. In this regard, two recent study protocols for randomized controlled trials on the use of an eHealth system for partners of patients with HNC are promising [44, 45]. However, further studies will be needed to test whether the proposed supportive care interventions are feasible.

## Conclusions

Caregivers of head and neck cancer (HNC) patients have a high need for supportive care. From diagnosis until 6 months after treatment, most of the unmet needs were in the “healthcare & illness” domain. The unmet needs decreased over time, but even 2 years after treatment, unmet needs were still being reported by more than a quarter of the informal caregivers. The unmet needs were statistically associated with both caregiver-related and patient-related variables. By identifying the changes in unmet supportive care needs at different points during the long-term follow-up period, and establishing subgroups that are prone to specific unmet needs, our results offer valuable insights for clinical practice. It is important to optimally support informal caregivers by involving them in patient counseling, providing them with relevant and understandable information, and referring vulnerable caregivers for (psychological) support.

## Supplementary Information

Below is the link to the electronic supplementary material.

Supplementary file1 (PDF 191 KB)

Supplementary file2 (PDF 343 KB)

## Data Availability

Data is not available as it is part of the ongoing multicenter cohort study NET-QUBIC.
