# Supplementary material for: Unmet supportive care needs among informal caregivers of patients with head and neck cancer in the first 2 years after diagnosis and treatment: a prospective cohort study
Source: Support Care Cancer. 2023 Apr 13;31(5):262. doi: 10.1007/s00520-023-07670-1 (PMC10101897; doi:10.1007/s00520-023-07670-1)

Appendix A: Flowchart of all eligible HNC patients and caregivers and reasons for non-participation

Also published in our previous article van Hof et al. [18].

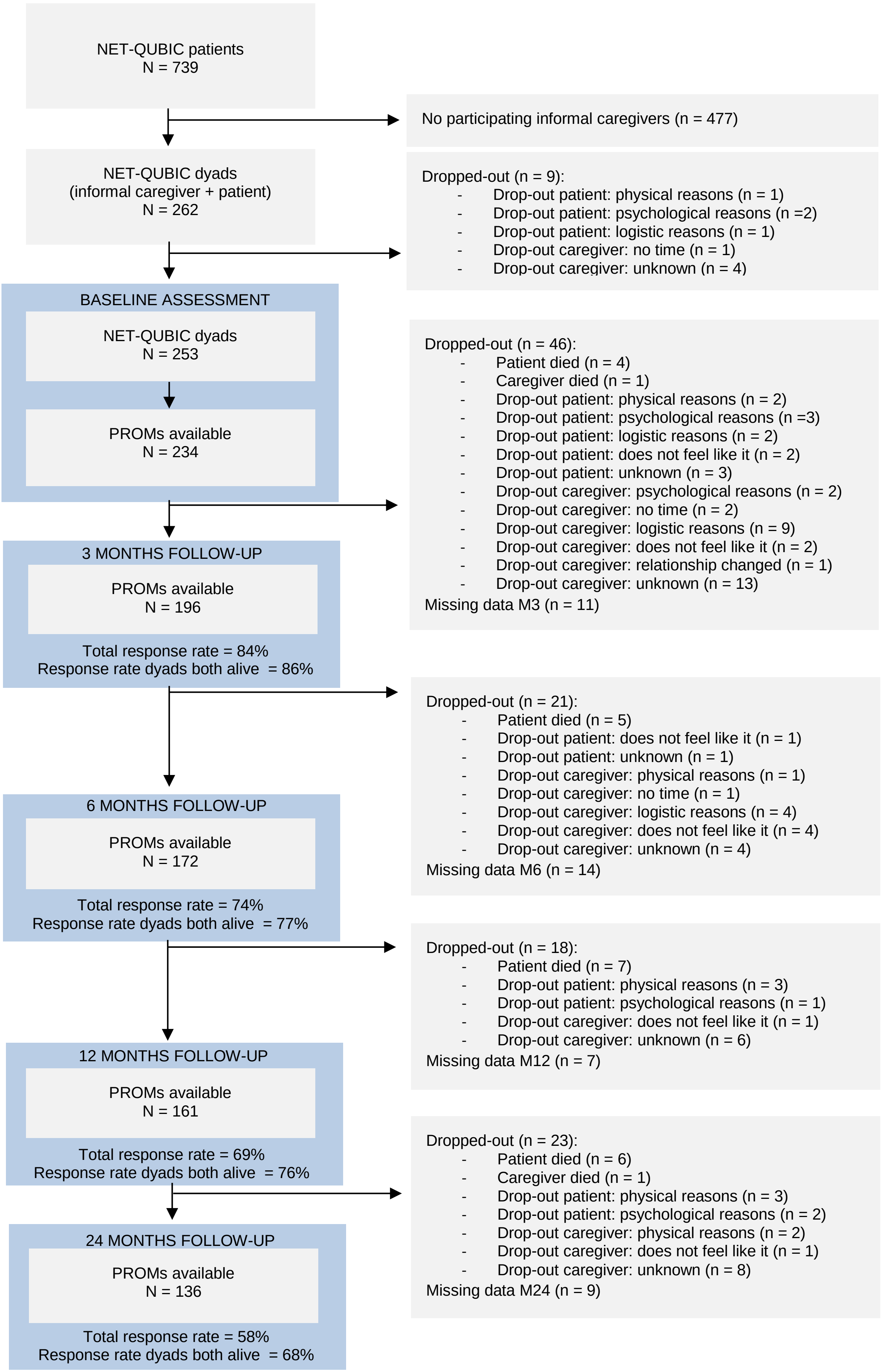

Supplement: Supplementary file 1 — Supplementary file1 (PDF 191 KB) [file 520_2023_7670_MOESM1_ESM.pdf]
