# Supplementary material for: Unmet supportive care needs among informal caregivers of patients with head and neck cancer in the first 2 years after diagnosis and treatment: a prospective cohort study
Source: Support Care Cancer. 2023 Apr 13;31(5):262. doi: 10.1007/s00520-023-07670-1 (PMC10101897; doi:10.1007/s00520-023-07670-1)
Supplement: Supplementary file 2 — Supplementary file2 (PDF 343 KB) [file 520_2023_7670_MOESM2_ESM.pdf]

Appendix B: Full table of variables associated with unmet needs in caregivers

Supportive Care Needs	Variable baseline		Estimate (95% CI)	P-value
**Emotional & relational**	Gender (C)	Male	“	
		Female	-.23 (-5.39 – 4.96)	0.925
	Age (C)		0.02 (-.21 – 0.26)	0.865
	Education (C)	Low	“	
		Intermediate	0.97 (-4.33 – 6.25)	0.715
		High	-1.26 (-6.67 – 4.25)	0.642
	Caregiver type (C)	Other	“	
		Spouse	3.43 (-7.9 – 14.56)	0.547
		Son/daughter	-.76 (-13.50 – 11.78)	0.907
	Anxiety (C)		0.25 (-.67 – 1.14)	0.589
	Depression (C)		0.89 (-.08 – 1.88)	0.073
	Global QoL (C)		0.02 (-.16 – 0.21)	0.796
	Social functioning (C)		-.06 (-.26 – 0.13)	0.521
	Physical functioning (C)		-.00 (-.19 – 0.18)	0.956
	Self-esteem (C)		-1.38 (-5.17 – 2.37)	0.472
	Disrupted schedule (C)		0.65 (-2.68 – 4.02)	0.698
	Family support (C)		0.29 (-3.01 – 3.60)	0.869
	Financial problems (C)		0.74 (-2.17 – 3.65)	0.615
	Health problems (C)		3.99 (-.69 – 8.58)	0.092
	Tumor stage (P)	I	“	
		II	1.56 (-4.64 – 7.82)	0.627
		III	9.01 (2.23 – 15.92)	0.010*
		IV	5.27 (-.02 – 10.65)	0.051
	Comorbidity (P)	None	“	
		Mild	5.36 (-.07 – 10.83)	0.054
		Moderate	7.52 (0.84 – 14.13)	0.030*
		Severe	3.93 (-4.02 – 11.92)	0.333
	WHO stage (P)	0	“	
		I/II	1.79 (-3.26 – 6.91)	0.477
	Unmet needs (P)		0.45 (0.12 – 0.78)	0.008*
	Time (months)		-.58 (-.69 - -.48)	<0.001*
**Health Care & illness**	Gender (C)	Male	“	
		Female	-3.29 (-10.03 – 3.45)	0.340
	Age (C)		0.28 (-.03 – 0.58)	0.076
	Education (C)	Low	“	
		Intermediate	-.47 (-7.53 – 6.45)	0.896
		High	-3.15 (-10.19 – 3.99)	0.388
	Caregiver type (C)	Other	“	
		Spouse	3.11 (-11.59 – 17.68)	0.670
		Son/daughter	7.24 (-9.16 – 23.75)	0.389
	Anxiety (C)		-.29 (-1.47 – 0.89)	0.614
	Depression (C)		1.12 (-.17 – 2.39)	0.090
	Global QoL (C)		0.03 (-.21 – 0.27)	0.821
	Social functioning (C)		-.02 (-.28 – 0.23)	0.885
	Physical functioning (C)		-.10 (-.33 – 0.14)	0.418
	Self-esteem (C)		0.42 (-4.58 – 5.48)	0.876
	Disrupted schedule (C)		1.88 (-2.51 – 6.35)	0.405
	Family support (C)		1.54 (-2.70 – 5.78)	0.479
	Financial problems (C)		0.46 (-3.35 – 4.31)	0.811
	Health problems (C)		2.53 (-3.66 – 8.64)	0.416
	Tumor stage (P)	I	“	
		II	6.43 (-1.80 – 14.55)	0.126
		III	10.09 (1.26 – 19.96)	0.024*
		IV	9.83 (2.96 – 16.74)	0.005*
	Comorbidity (P)	None	“	
		Mild	5.02 (-2.15 – 12.19)	0.167
		Moderate	6.35 (-2.42 – 15.21)	0.155
		Severe	0.96 (-9.48 – 11.34)	0.852
	WHO stage (P)	0	“	
		I/II	3.42 (-3.14 – 10.12)	0.312
	Unmet needs (P)		0.41 (-.02 – 0.82)	0.060
	Time (months)		-1.20 (-1.38 - -1.05)	<0.001*
**Practical**	Gender (C)	Male	“	
		Female	-3.14 (-8.04 – 1.70)	0.196
	Age (C)		0.08 (-.14 – 0.30)	0.463
	Education (C)	Low	“	
		Intermediate	0.34 (-4.64 – 5.19)	0.894
		High	-2.71 (-7.63 – 2.38)	0.289
	Caregiver type (C)	Other	“	
		Spouse	-1.41 (-12.10 – 9.03)	0.793
		Son/daughter	0.53 (-11.40 - 12.34)	0.931

	Anxiety (C)		-.32 (-1.15 – 0.54)	0.452
	Depression (C)		0.87 (-.01 – 1.77)	0.063
	Global QoL (C)		0.17 (0.00 – 0.34)	0.049*
	Social functioning (C)		0.03 (-.15 – 0.21)	0.714
	Physical functioning (C)		-.16 (-.33 – 0.01)	0.058
	Self-esteem (C)		-.47 (-4.06 – 3.13)	0.794
	Disrupted schedule (C)		-1.31 (-4.63 – 1.84)	0.422
	Family support (C)		1.25 (-1.78 – 4.34)	0.430
	Financial problems (C)		4.04 (1.30 – 6.83)	0.003*
	Health problems (C)		3.28 (-1.24 – 7.87)	0.159
	Tumor stage (P)	I	“	
		II	4.50 (-1.33 – 10.40)	0.131
		III	8.68 (2.33 – 15.07)	0.006*
		IV	6.04 (1.09 – 11.04)	0.017*
	Comorbidity (P)	None	“	
		Mild	1.64 (-3.40 – 6.71)	0.528
		Moderate	4.38 (-1.85 – 10.62)	0.162
		Severe	-2.00 (-9.31 – 5.37)	0.595
	WHO stage (P)	0	“	
		I/II	2.92 (-1.88 – 7.74)	0.230
	Unmet needs (P)		0.19 (-.15 – 0.54)	0.277
	Time (months)		-.37 (-.50 - -.26)	<0.001*
**Work & social**	Gender (C)	Male	“	
		Female	-1.68 (-6.57 – 3.15)	0.498
	Age (C)		-.02 (-.24 – 0.20)	0.852
	Education (C)	Low	“	
		Intermediate	0.74 (-4.13 – 5.67)	0.768
		High	-2.65 (-7.64 – 2.25)	0.297
	Caregiver type (C)	Other	“	
		Spouse	-1.05 (-11.75 – 9.59)	0.847
		Son/daughter	-1.17 (-13.12 – 10.67)	0.852
	Anxiety (C)		-.12 (-.97 – 0.72)	0.777
	Depression (C)		0.88 (-.04 – 1.80)	0.060
	Global QoL (C)		-.02 (-.19 – 0.15)	0.795
	Social functioning (C)		-.06 (-.24 – 0.13)	0.528
	Physical functioning (C)		0.04 (-.13 – 0.21)	0.634
	Self-esteem (C)		-1.73 (-5.29 – 1.83)	0.338
	Disrupted schedule (C)		0.79 (-2.35 – 3.94)	0.621
	Family support (C)		0.29 (-2.76 – 3.30)	0.849
	Financial problems (C)		1.30 (-1.44 – 4.00)	0.348
	Health problems (C)		1.54 (-2.89 – 5.96)	0.488
	Tumor stage (P)	I	“	
		II	3.49 (-2.45 – 9.38)	0.245
		III	6.48 (0.13 – 12.9)	0.045*
		IV	4.33 (-.64 – 9.31)	0.087
	Comorbidity (P)	None	“	
		Mild	4.20 (0.88 – 9.23)	0.106
		Moderate	6.07 (-.09 – 12.30)	0.054
		Severe	0.20 (-7.32 – 7.72)	0.954
	WHO stage (P)	0	“	
		I/II	2.60 (-2.20 – 7.33)	0.286
	Unmet needs (P)		0.40 (0.07 – 0.72)	0.019*
	Time (months)		-.59 (-.71 -.46)	<0.001*

Linear mixed models were adjusted for gender, age, education level, caregiver type, tumor stage, WHO-status and total unmet needs patient (baseline), caregiver burden (baseline), anxiety caregiver (baseline), depression caregiver (baseline), physical functioning (baseline) and social functioning (baseline). (C) = Caregiver, (P) = Patient.
